# The future of trauma care in Germany 2030: challenges, opportunities, and strategic directions

**DOI:** 10.1007/s00068-026-03102-x

**Published:** 2026-03-12

**Authors:** Christopher Spering, Michaela Lemm, Sabine Finke, Malina Wrobel, Alexander Haering, Axel Franke, Kai Sprengel, Ricarda Seemann, Dan Bieler

**Affiliations:** 1 German Society of Trauma Surgery (DGU), Berlin, Germany; 2 Institute for Healthcare Business GmbH, Essen, Germany; 3 RWI – Leibniz-Institute for Economic Research, Essen, Germany; 4 Department of Trauma Surgery, Orthopedics and Plastic Surgery, Universitätsmedizin Göttingen, Robert-Koch-Str. 40, D-37075 Göttingen, Germany

## Abstract

**Background:**

Trauma care in Germany is a critical component of public healthcare, encompassing preclinical to rehabilitative phases of patient management. Demographic shifts, workforce shortages, and increasing patient complexity challenge the sustainability of trauma care. This study aims to analyze current trauma care structures, forecast future demands, and propose strategies to secure high-quality trauma care in Germany by 2030.

**Methods:**

A mixed-methods approach was employed, combining a quantitative analysis of national hospital and ICD-coded diagnostic data (2010–2019) to model infrastructure, analyze patient volumes, and forecast demand to 2030 under different scenarios. This was supplemented with an online survey of 752 trauma surgeons nationwide to assess perceptions of future challenges and professional satisfaction, and a structured expert panel with 23 experts to qualitatively interpret the data and develop strategic recommendations.

**Results:**

Survey respondents anticipate a significant rise in age-related trauma cases and a worsening shortage of both medical and non-medical staff. Hospital infrastructure is unevenly distributed, with particular accessibility issues in rural and eastern regions of Germany. Forecasts predict increasing trauma cases in older adults and a reduction in hospital beds, especially in rural areas. The data analysis identified critical areas for intervention including improved training, telemedicine integration, centralized trauma centers, and enhanced image and attractiveness of the trauma surgeon profession.

**Conclusions:**

Ensuring sustainable trauma care in Germany requires structural reforms, sectoral integration, and workforce development. Policy must support case-number independent resource orientated financing, sector-crossing cooperation, and the incorporation of advanced digital technologies. Centralized specialization combined with broad generalist competencies in the field will be essential to meet future demands.

## Introduction

Trauma care in Germany embodies a critical public service, pledging comprehensive care for accident victims of all severities – from minor injuries to complex polytrauma – with an emphasis on restoring patient functionality and socio-economic reintegration. Despite its importance, trauma care faces mounting pressures. The continuum encompasses prevention, pre-hospital trauma care, emergency department treatment, operative and non-operative therapy, inpatient care, rehabilitation, and long-term follow-up aimed at reintegrating patients into their social and economic environments. However, demographic changes – including the retirement of the large baby boomer generation – and evolving healthcare demands pose significant challenges. The workforce is shrinking, the complexity of patient cases is rising, and current structures risk becoming inefficient or unsustainable. Concurrently, the profession of trauma surgery struggles with image and recruitment challenges, compounded by rising documentation demands and evolving expectations for work-life balance.

This study presents findings from a collaborative project by the German Society for Trauma Surgery (DGU), RWI Leibniz Institute, and Institute for Health Care Business GmbH aimed at presenting a detailed analysis of these challenges and anticipating future developments as well as outlining evidence-based strategies and formulating strategic responses to maintain and enhance trauma care quality beyond 2030.

## Methods

A prospective, mixed-methods approach was employed to analyze the current state and future trajectory of trauma care in Germany up to the year 2030. This comprehensive study was conducted as a collaborative project between the German Society for Trauma Surgery (DGU), the RWI – Leibniz-Institute for Economic Research, and the Institute for Health Care Business GmbH. The methodology consisted of three core components: (1) a quantitative analysis of national hospital and diagnostic data (2), a nationwide online survey of trauma surgeons, and (3) a structured expert panel process. 

### Quantitative data analysis

A comprehensive quantitative analysis was performed to model the existing trauma care infrastructure, map patient volumes, and project future demand to the year 2030. This analysis formed the empirical and scientific foundation of the study and was based on several national data sources:

- **Hospital Quality Reports** from 2014 to 2019, as mandated by the Federal Joint Committee (G-BA).
- **Deep-dive ICD-coded diagnostic data** from the Federal Statistical Office for the years 2010 to 2019.
- **Hospital Directories** from the statistical offices of the federal and state governments for the years 2010 to 2019.
- **Population Data and Projections** Growth from Knowledge (GfK, 2019) and the Federal Statistical Office (Destatis; 14th Coordinated Population Projection).

The analysis was structured into three main parts: *infrastructure and accessibility*, *patient volume and case mix*, and *future projections*.

- **Infrastructure and Accessibility Analysis**: The stability of the hospital landscape was evaluated by classifying all German districts into “stable,” “labile,” or “unstable” categories. An “unstable” classification was assigned to districts where either the average number of beds per hospital site was 175 or fewer, or where at least 50% of hospital sites were small (≤ 150 beds). A longitudinal analysis tracked the change in trauma-specific bed capacity from 2010 to 2019, comparing urban and rural regions. Furthermore, a geospatial analysis was conducted to map the 30-minute travel time accessibility of trauma centers. This included a simulation modeling the impact on accessibility that would result from the closure of all trauma units in small hospitals (< 200 beds), with a particular focus on eastern Germany.
- **Patient Volume and Case Mix Analysis**: Using the deep-dive diagnostic data from 2010 to 2019, inpatient case numbers were analyzed to identify longitudinal trends in patient demographics and injury types. Cases were grouped into nine distinct trauma clusters based on ICD-10 codes: head/brain trauma, spinal injuries, upper extremity injuries, hand injuries, lower extremity injuries, foot injuries, polytrauma, endoprosthetics, and other injuries. A specific sub-analysis focused on the development of geriatric trauma cases (defined by ICD codes S32, S42, S72) and their distribution across hospitals of different sizes (small, medium, large) between 2014 and 2019.
- **Future Projections to 2030**: Projections of inpatient case volumes for each trauma cluster were calculated from the 2019 baseline to 2030 under two distinct scenarios. The first scenario projected changes based solely on demographic developments. The second, more complex scenario incorporated the demographic projection while also adjusting for the potential shift of inpatient cases to ambulatory care settings (incl. amb. potential), reflecting ongoing healthcare policy trends.

All quantitative analyses and projections are based on data up to and including 2019. Consequently, structural disruptions associated with the COVID-19 pandemic and subsequent health-policy measures, including ongoing hospital reform initiatives and inflation-related cost pressures, are only partially captured in the model. The projections should therefore be interpreted as scenario-based estimates on a pre-pandemic baseline rather than as deterministic forecasts.

### Online survey

An online survey was conducted among the members of the DGU to assess their perceptions of current and future professional environments. A total of 752 DGU members participated. The respondent cohort was composed primarily of senior surgeons (38.3%) and chief surgeons (26.6%), with a majority being male (79.9%) and within the 41–60 year age range (62.0). Participants were asked to rate the probability of specific future developments and their perceived impact on daily professional life using Likert scales ranging from 0 (highly improbable/no impact) to 5 (highly probable/very high impact). Additionally, overall job satisfaction was rated on a scale of 0 to 10.

In the survey, ‘non-medical personnel’ referred to non-physician staff involved in trauma care, including nursing staff, physiotherapists, operating room assistants, and administrative or support staff directly contributing to patient care processes.

Structured Expert Panel Process

### Structured expert panel process

A two-day structured expert panel was held on May 25–26, 2022, in Ulm, with 23 expert members of the DGU. The objective was to qualitatively interpret the quantitative findings, explore future challenges, and formulate strategic goals. Participants were divided into three thematic working groups: (1) Assessment, Motivation, and Continuing Education; (2) Complexity of Future Trauma Care; and (3) Image of the Trauma Surgeon. The results from each working group were synthesized and presented in a plenary session for final discussion and consolidation.

## Results

### A system in transformation: quantitative analysis of infrastructure and patient demographics

Our quantitative analysis, based on national hospital and diagnostic data from 2010 to 2019 with projections to 2030, reveals a trauma care system undergoing a profound structural and demographic transformation.

### Infrastructure under strain and disparate access

**Fig. 1:**
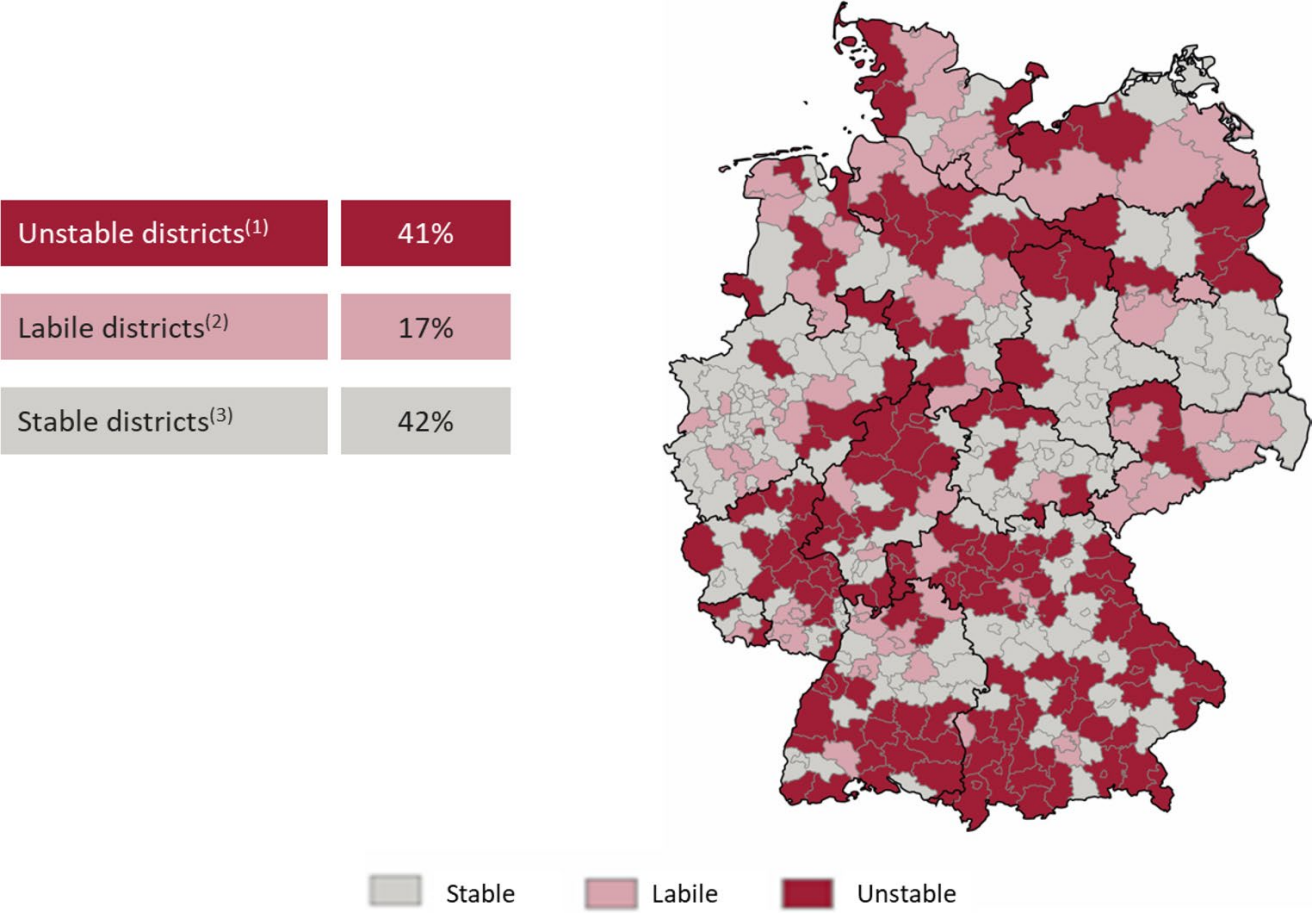
Map of Germany showing the stability of hospital structures by district. The map categorizes districts into three groups based on hospital bed counts and size of hospital sites: (1) Unstable districts – Districts where either the average number of beds per hospital site is at most 175, or where at least 50% of hospital sites are small (≤ 150 beds). These districts are marked with a certain color (not specified in the text) (2) Labile districts)– Districts without the above criteria but with at least one very small hospital site (≤ 100 beds) (3) Stable districts – All other districts. The map shows that 41% of districts fall into the unstable category, 17% into labile, and 42% into stable. Additionally, the map combines independent cities with their surrounding rural districts for analysis. Note: the map does not consider competitive intensity, which may affect stability especially in densely populated urban areas. This visualization highlights regional disparities in trauma care infrastructure, emphasizing the presence of many small or low-capacity hospitals, particularly in rural areas, which potentially threatens the stability and accessibility of trauma care services

**Fig. 2:**
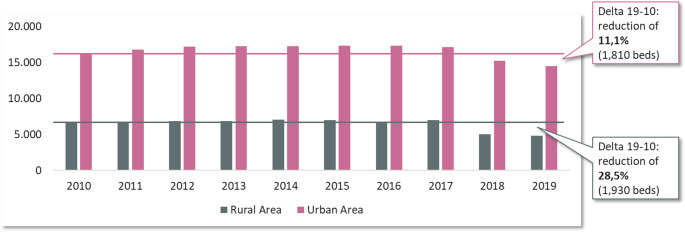
Development of trauma beds in urban vs. rural areas (2010–2019). The amount of Trauma Beds in urban vs. rural areas over the time period of2010 to 2019. Urban trauma beds decline approximately 11.1%, rural trauma beds decline about 28.5%. The x-axis is years, y-axis is number of beds. Rural columns show steeper decline

**Fig. 3:**
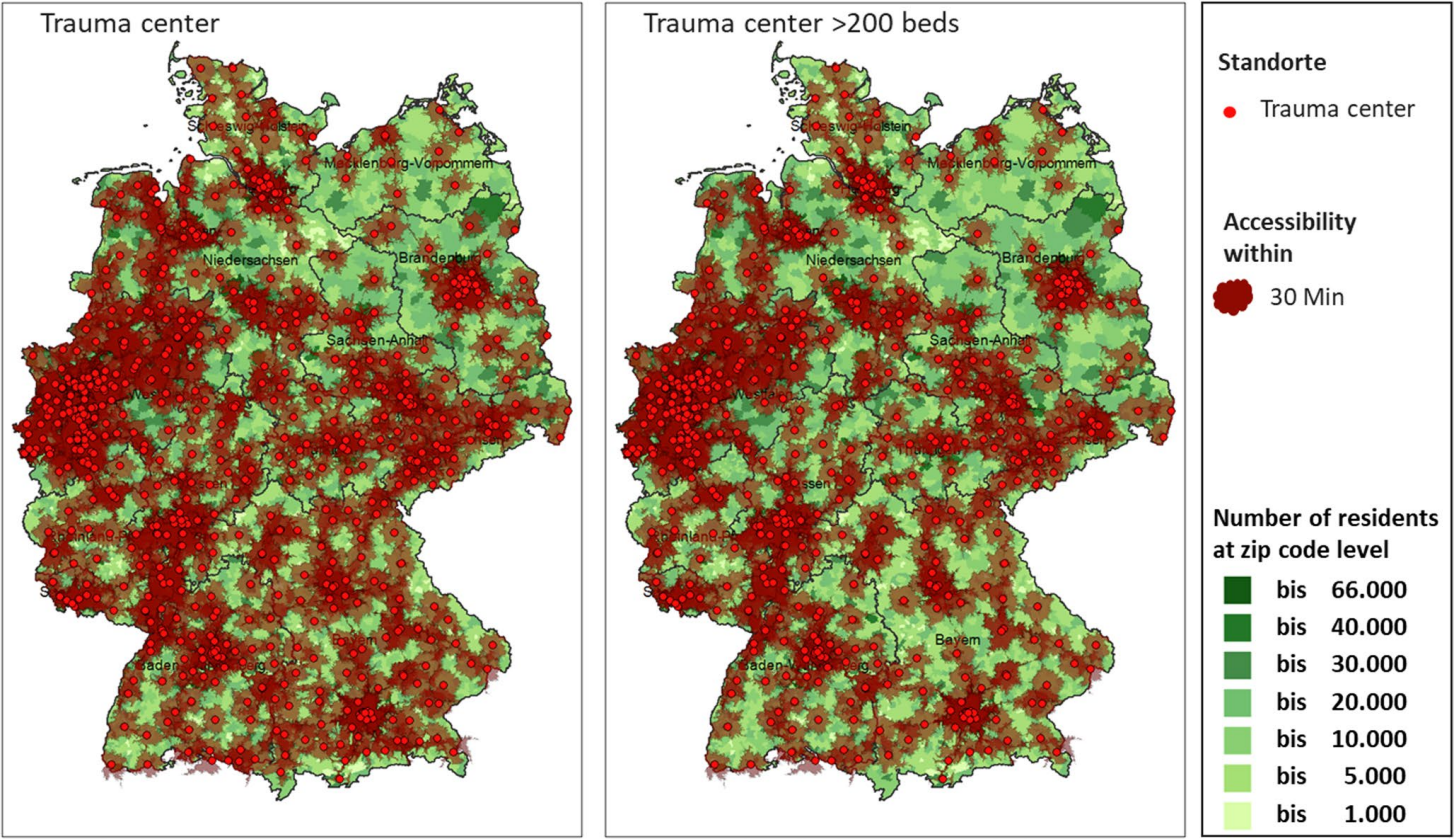
Map with data on trauma centers in Germany, focusing on their size and the presence of specialized departments in trauma surgery. On the left side, the map shows 646 locations with trauma care, among which 203 do not have separately designated trauma surgery and/or orthopedic departments but only general surgery. Of these 203 locations, 22 are small, 143 are mid-size, and 38 are large facilities. On the right side, the figure highlights 530 trauma centers with more than 200 beds. Among these larger centers, 398 have departments specialized in trauma surgery and/or orthopedics, and 513 have general surgery departments. The figure also includes a representation of population numbers on the postal code, categorized by ranges (up to 66,000; up to 40,000; down to as low as 1,000 inhabitants) and shows the accessibility of trauma centers within a 30-minute travel time. This visualization emphasizes the distribution of trauma care facilities, the level of specialization available, and the reachability of these centers for the population, which is critical for emergency care planning

The German hospital landscape exhibits significant structural vulnerabilities. 41% of all districts were classified as having “unstable” hospital structures, characterized by either a low average number of beds per site (≤ 175) or a high proportion of small facilities (≥ 50% of sites with ≤ 150 beds). This instability is most pronounced in rural and eastern German regions (Fig. 1). This structural fragility is exacerbated by a sharp decline in capacity, particularly in rural areas, which saw a 28.5% reduction in trauma surgery beds between 2010 and 2019, compared to an 11.1% reduction in urban centers (Fig. 2). Geospatial modeling confirms that the potential closure of trauma units in small hospitals (< 200 beds) would critically jeopardize 30-minute travel time accessibility, creating significant gaps in emergency coverage, especially in eastern Germany (Fig. 3).

## Economic and demographic pressure on the German hospital system

**Fig. 4:**
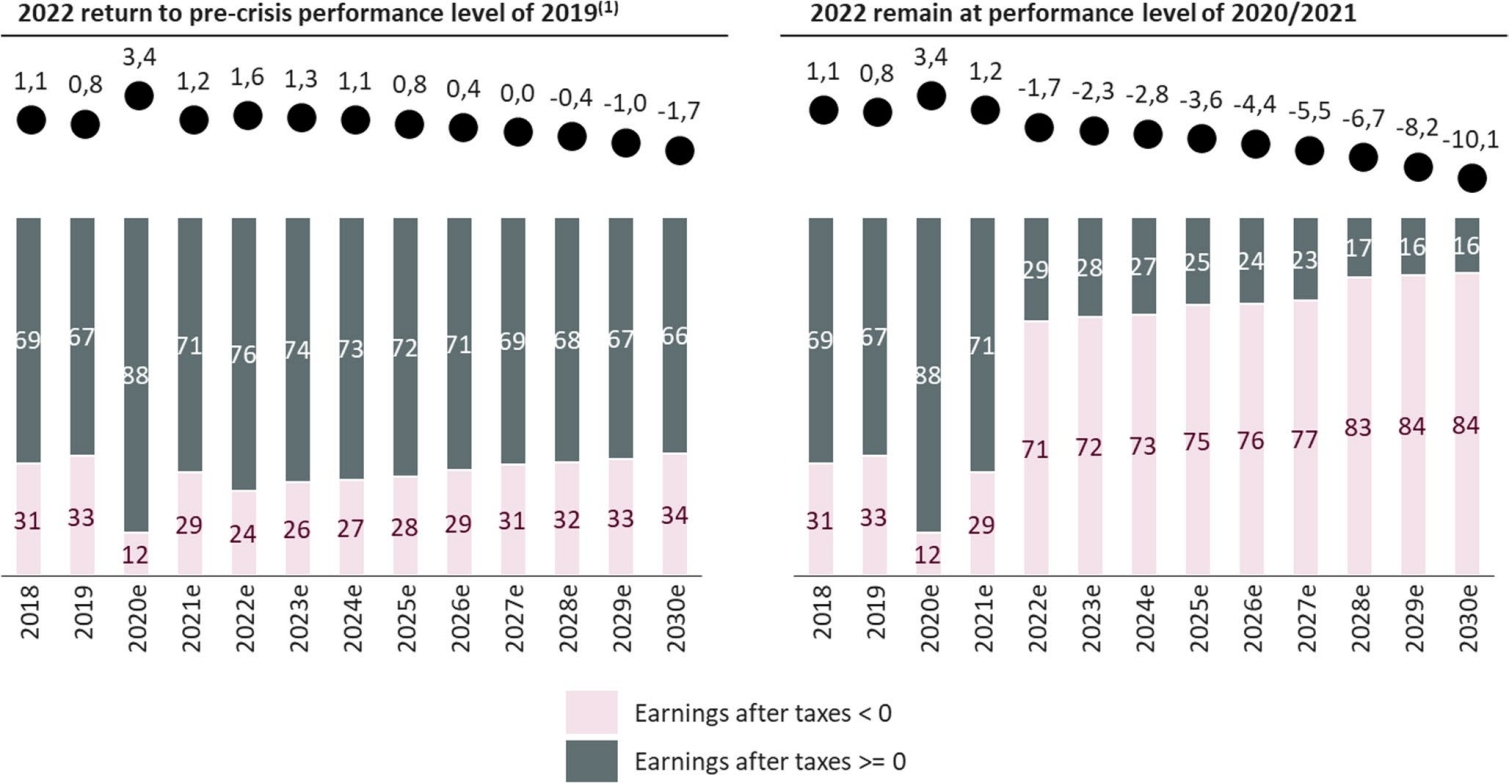
Illustration of the strained economic situation of hospitals in Germany (2018–2030e) under two calculation models. It shows the relationship between case numbers and the financial status of hospitals. The figure projects that even if case numbers were to return to the 2019 pre-crisis level, about one-third of hospitals would still have a negative annual financial result. If case numbers were to remain at the lower levels seen during 2020/2021, more than half of the hospitals would likely face negative annual results. This projection does not yet take into account increased energy costs and general price inflation

**Fig. 5:**
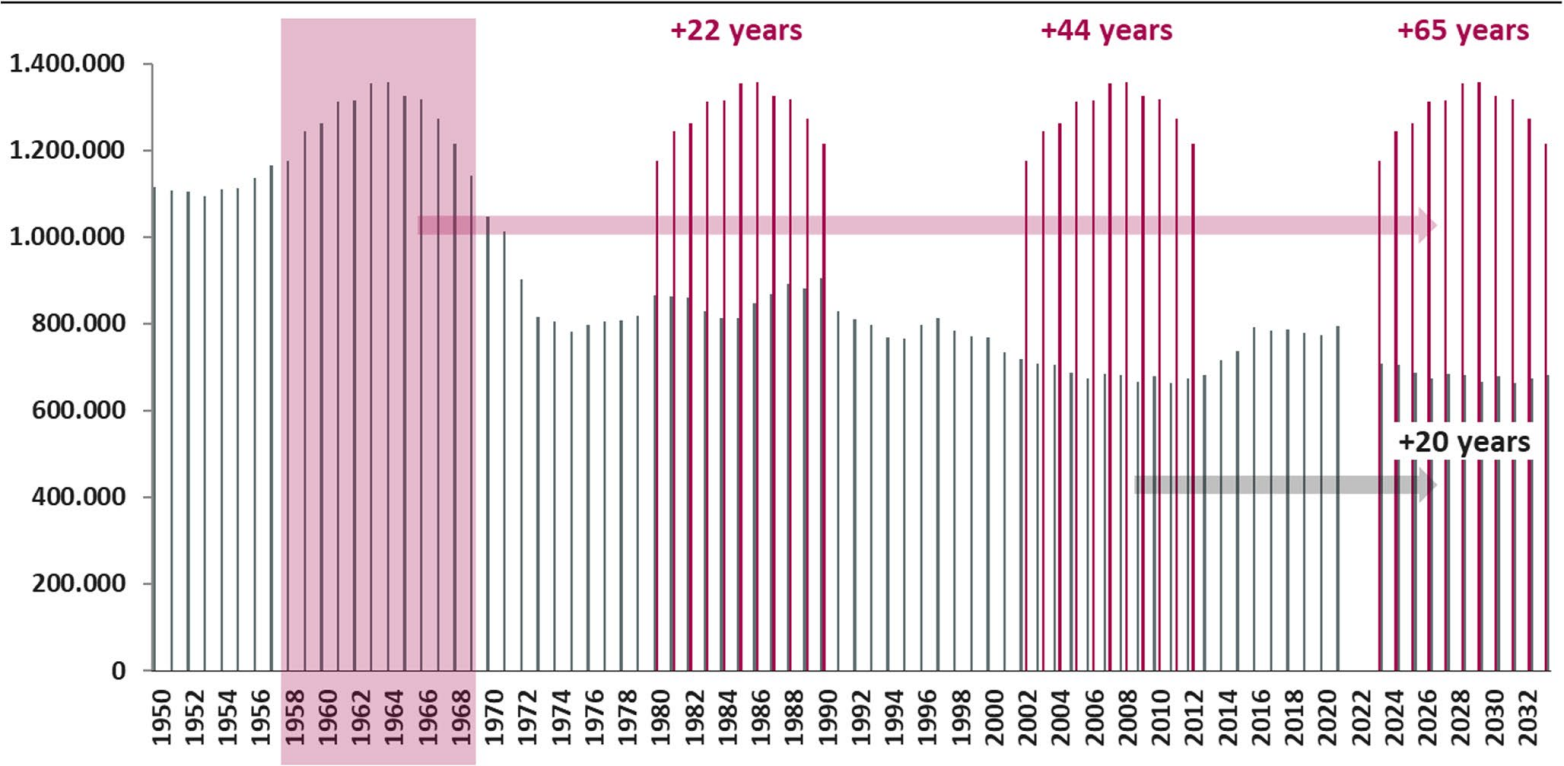
Number of births in Germany (East and West) from 1950 to 2032 with regard to the Baby-Boomer generations path to retirement (2024–2032). The trend over time highlights the demographic changes in the population. It illustrates that by the year 2023, there will be approximately 1.2 million people aged 65 and older, which is almost double the number of 20-year-olds (around 700,000). This demographic shift is significant for the challenges faced in trauma surgery and healthcare planning, as it leads to an increase in geriatric trauma injuries and associated complexities as well as a simultaneous lack of workforce due to retirement of the baby-boomer generation starting in 2024

The analysis identified significant economic and demographic pressure that fundamentally challenge the future stability and capacity of trauma care in Germany (Figs. 4 and 5).

The financial viability of the German hospital landscape is precarious, heavily dependent on patient case volumes. Projections modeling the economic situation of hospitals until 2030 reveal a strained outlook under two different scenarios (Fig. 4). In an optimistic model where case numbers return to the pre-crisis levels of 2019, it is projected that about one-third of hospitals would still operate with a negative annual financial result. However, if case numbers were to stagnate at the lower levels seen during the COVID-19 pandemic years of 2020/2021, more than half of all hospitals would likely face negative annual results. It is important to note that these forecasts do not yet factor in the recent considerable increases in energy costs and general price inflation, suggesting the actual financial situation could be even more critical.

Compounding these economic vulnerabilities is a major demographic shift, characterized by the impending retirement of the “Baby-Boomer” generation from 2024 to 2032 (Fig. 5). This trend exerts a dual pressure on the healthcare system: a simultaneous reduction in the available workforce and an increase in patient demand and complexity. Analysis of population trends illustrates that by 2023, there were approximately 1.2 million people aged 65, a figure nearly double the number of 20-year-olds (around 700,000) in the population. This demographic imbalance is projected to exacerbate workforce shortages across all sectors, including healthcare, while the aging population will drive a significant increase in geriatric trauma injuries and associated clinical complexities.

## A shifting patient population and divergent case volume projections

**Fig. 6:**
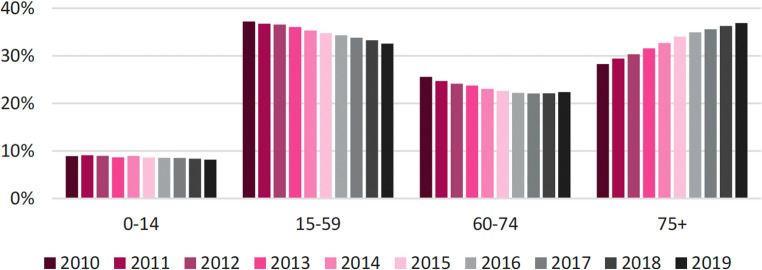
Development of traumatological case numbers by age group from 2010 to 2019. The proportion of trauma cases has decreased in the age groups from 15 to 74 years but increased in the age group of 75 years and older. This suggests a shift in the demographic distribution of trauma patients over this period, with an increasing share of older patients requiring trauma care

**Fig. 7:**
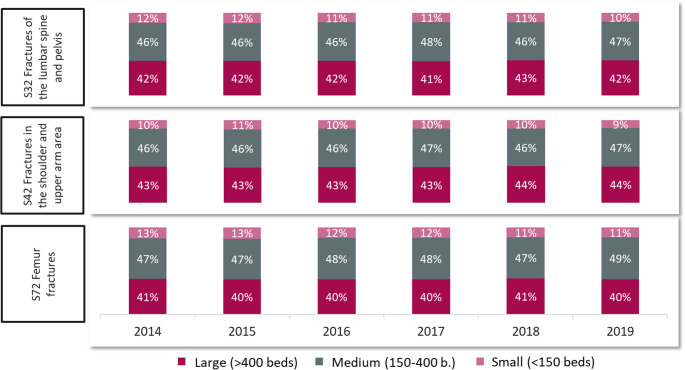
Development of case numbers for geriatric trauma injuries from 2014 to 2019, by hospital size categories. The figure specifically considers fractures of the lumbar spine and pelvis (S32), fractures in the shoulder and upper arm area (S42), and femur fractures (S72) treated in the departments of general surgery, trauma surgery, orthopedics, and geriatrics. The figure illustrates that 85% to 90% of geriatric trauma injuries are treated in large and medium-sized hospitals, with only minor changes from 2014 to 2019. Despite the overall increase in case numbers that corresponds to demographic changes, the hospital care structures for trauma patients in Germany show a contrasting trend. There is a movement toward centralization of care, but many regions rely on unstable hospital structures (Fig. 1). This points to emerging qualitative deficits in trauma care, especially in rural areas, where interdisciplinary emergency care with minimal waiting times is crucial to prevent secondary complications such as pneumonia or wound healing disorders

The patient demographic has shifted decisively towards older age groups. Between 2010 and 2019, the proportion of trauma cases increased steadily among patients aged 75 and older, while concurrently decreasing across all age groups from 15 to 74 years (Fig. 6). A trend towards centralization for complex geriatric cases is already evident, with 85–90% of specific geriatric trauma injuries (ICD codes S32, S42, S72) being treated in medium and large-sized hospitals between 2014 and 2019 (Fig. 7).

**Fig. 8:**
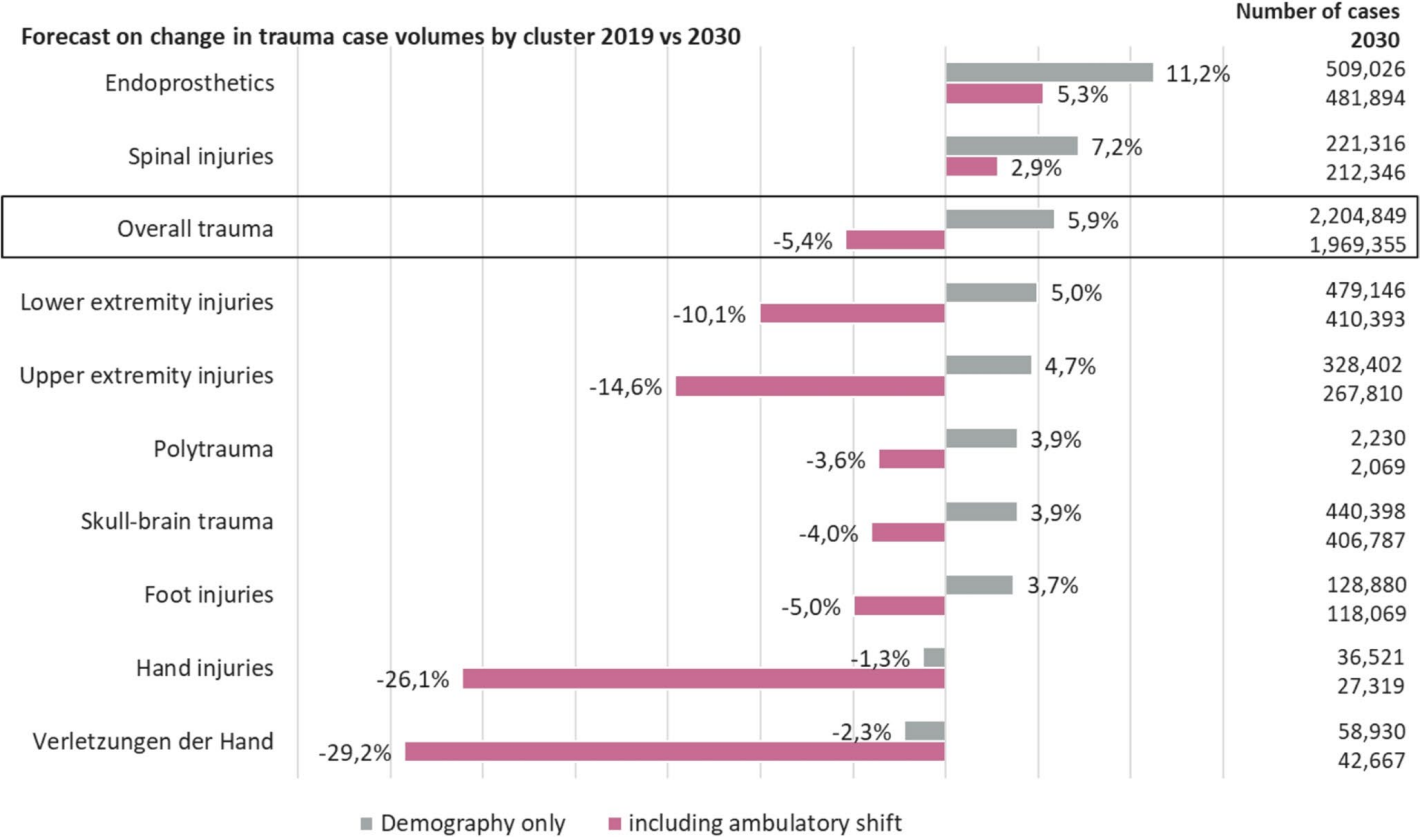
Forecast on change in trauma case volumes by cluster 2019 vs. 2030. Comparison of two scenarios: one considering only demographic developments and the other including the potential shift of cases to outpatient: (**a**) under demographic considerations alone, most traumatological clusters are expected to see an increase in case numbers by 2030 compared to 2019. Exceptions are injuries of the foot and hand, which are projected to decrease. (**b**) when factoring in the outpatient potential, there is a predicted reduction in inpatient case numbers across almost all clusters, with the largest decreases expected in injuries of the foot and hand. (**c**) inpatient case numbers are expected to increase only in (fracture) endoprosthetics and spinal injuries. Overall, this emphasizes a trend towards more outpatient treatments and a slight overall decrease in inpatient traumatology cases by about 5%, despite demographic-driven increases in total case numbers, especially in patients aged 75 and older

Projections of inpatient case volumes to 2030 reveal a complex future landscape shaped by two opposing forces (Fig. 8):

1. **Demographics Only Scenario**: Based solely on demographic aging, overall inpatient trauma cases are projected to increase by 5.9%. The primary drivers are endoprosthetics (+ 11.2%) and spinal injuries (+ 7.2%), while only hand (-2.3%) and foot (-1.3%) injuries are expected to decline.
2. **Ambulatory Shift Scenario**: When factoring in the anticipated shift of less complex cases to outpatient settings, overall inpatient trauma cases are projected to *decrease* by 5.4%. However, this aggregate figure masks a critical trend toward higher acuity. While numerous injury types are forecast to see steep inpatient declines (e.g., head/brain trauma − 29.2%; foot injuries − 26.1%), inpatient cases for spinal injuries are still projected to grow by 5.3%.

These divergent forecasts point to a future defined by fewer, but significantly more complex and resource-intensive, inpatient trauma cases (Fig. 8).

### Workforce perceptions: corroboration from the front lines

The online survey of 752 trauma surgeons (Tables 1 and 2) provided qualitative validation of the quantitative trends, revealing moderate job satisfaction (mean 6.6/10) alongside significant concerns. Surgeons rated the likelihood of an “increase in patients with fractures over 80 years” as highly probable (mean 4.7/5), directly aligning with the demographic data. The most significant impacts anticipated on their daily work were an “increase in geriatric trauma cases” (mean 4.3/5) and worsening “shortages of non-medical personnel” (4.2/5) and medical staff (4.0/5). These perceptions underscore the workforce’s acute awareness of the dual pressures from a changing patient population and systemic resource constraints. Additionally, an “increase in documentation workload” (mean likelihood 4.5/5) and the “implementation of a digital patient record” (mean impact 4.1/5) were identified as major administrative challenges compounding clinical pressures.

**Table 1:** Demographic and professional characteristics of survey participants

Characteristic	Percentage (%)
Senior Surgeons	38.3
Chief Surgeons	26.6
Male	79.9
Age 41–50	28.2
Age 51–60	33.8

**Table 2:** Top 5 expected challenges impacting trauma care (Survey mean scores 0–5)

Challenge	Mean Score
Increase in patients > 80 years	4.7
Societal aging	4.6
Increase in documentation workload	4.5
Electronic image processing	4.5
Introduction of digital patient record	4.4

### Strategic responses: insights from the expert panel

The structured expert panel synthesized the quantitative and survey data to formulate strategic goals for ensuring the future of trauma care:

- **Training for a New Reality**: Acknowledging that practical patient exposure is irreplaceable, yet threatened by declining case volumes, the panel advocated for a hybrid training model. This includes using simulations for basic skills, delegating non-operative tasks to maximize surgeons’ operative time, and establishing formal rotational training programs through existing networks like the TraumaNetzwerk DGU®. This strategy directly addresses the need to maintain surgical competence despite fewer cases per location.
- **A Dual Model for Complex Care**: To manage the rising complexity of geriatric trauma while ensuring broad access, experts proposed a two-tiered structural model: (1) centralization of highly specialized procedures (e.g., complex joint, spine, and pelvic reconstruction) in high-volume trauma centers, and (2) maintenance of strong generalist trauma competencies in peripheral hospitals. This model’s success hinges on the widespread integration of telemedicine and a clear definition of competencies for each level of care.
- **Rebuilding the Profession’s Image**: To counter recruitment challenges, the panel stressed the need to actively shape the trauma surgeon’s image. The goal is to move from a perception of an “invisible helper in need” to that of a visible, highly skilled expert and empathetic partner in restoring patients’ quality of life and mobility. Proposed tactics include targeted public relations campaigns, a stronger academic presence, and strategic use of social media to engage with the public and prospective trainees.

## Discussion

The German trauma care system faces a multifaceted transformation driven by demographic changes, workforce dynamics, and technological progress. The marked increase in geriatric trauma necessitates adaptation in clinical protocols and interdisciplinary care involving geriatrics, rehabilitation, and social services. Simultaneously, workforce shortages compel structural reforms including centralization and delegation to extend the reach of trauma surgeons.

The shift toward ambulatory care and digitalization presents both challenges and opportunities. Integrating sector-spanning budgets and collaborative care models can foster continuity and efficiency. The trauma surgeon’s role is evolving beyond operative expertise to encompass patient-centered coordination and leadership within complex care networks.

An important contextual factor is that our projections are anchored in pre-pandemic data up to 2019. Subsequent developments - most notably the COVID-19 pandemic, accelerated hospital consolidation, sharp increases in energy and personnel costs, and the current hospital reform process in Germany - are likely to intensify several of the trends we describe. Analyses from the Krankenhaus Rating Report already suggest that, even under optimistic case-number scenarios, a considerable share of hospitals will operate with negative annual results. It is therefore plausible that economic and structural pressures on trauma care will be even more pronounced than our baseline scenarios indicate. Nevertheless, the central qualitative findings of our study - aging of the trauma population, increasing case complexity, vulnerability of rural infrastructure, and workforce shortages - are robust to these recent shocks and are in line with current national planning debates.

Our recommendation to maintain approximately 700–800 trauma care sites with 30-minute accessibility aligns with the current German hospital reform debate, which seeks to combine structural consolidation with guaranteed regional access. The centralization of highly specialized procedures and the designation of different levels of care proposed in our framework are consistent with the emerging level-of-care concept, while our emphasis on case-number independent baseline funding directly addresses the challenge of financing high-readiness, low-volume trauma services under DRG-dominated reimbursement.

Addressing the decline in trauma surgeon attractiveness requires improvements in work-life balance, reduction of bureaucratic burdens, and enhanced training pathways emphasizing competence and motivation. A positive public image reinforced by transparent communication and role modeling can support recruitment and retention.

## Conclusions

The future of trauma care in Germany demands proactive, coordinated action to address demographic, economic, and workforce challenges. By restructuring care delivery, integrating sectors, modernizing education, and improving the trauma surgeon’s image, sustainable, high-quality trauma care can be secured for 2030 and beyond. Political commitment and adaptive policy frameworks are essential to realize these goals.

To secure high quality trauma care in Germany by 2030 and beyond, comprehensive measures are required:

- Maintain 700 trauma care centers nationwide, ensuring 30-minute accessibility via digital and telemedical support. This target range is based on our geospatial accessibility analyses (30-minute travel time) and simulations of the effects of closing trauma units in smaller hospitals (< 200 beds), particularly in rural and eastern regions.
- Implement case-number independent resources orientated funding to sustain infrastructure.
- Foster sector-overlapping treatment and billing to enable flexible use of scarce personnel.
- Enhance education with competency-based curricula, simulation, and intersectoral rotations.
- Promote the trauma surgeon profession through targeted public relations and work environment reforms.

These steps will help Germany meet the growing demands of an aging population while ensuring sustainable, high-quality trauma care.

### Recommendations

Based on the findings and conclusion of the study, the following recommendations are proposed:

3. **Structural Optimization**: Maintain 700–800 trauma care sites nationwide, leveraging telemedicine to ensure accessibility. Introduce case-number independent resource orientated baseline funding to secure readiness.
4. **Patient Flow and Triage**: Establish integrated emergency centers combining outpatient and inpatient emergency services, supported by validated triage systems and enhanced public guidance on emergency use.
5. **Sector Integration**: Enable cross-sector treatment and billing, including ambulatory surgery centers linked with trauma networks. Facilitate cross-sectoral training programs.
6. **Workforce and Education**: Centralize trauma care to allow specialization while ensuring generalist coverage in the periphery. Empower new healthcare roles to alleviate physician workload. Reform training to be competency-based with emphasis on practical exposure, communication, and leadership skills. Enhance the profession’s image to improve recruitment and retention.

## Summary

Trauma care in Germany is on the brink of significant transformation. The ultimate goal of a high-quality trauma care system is not merely to address the injury itself, but to restore impaired function. The restoration of quality of life and mobility is the core objective of modern and forward-orientated trauma surgery care. Achieving this requires fundamental structural changes within an otherwise stable healthcare system.

In addition to ensuring robust initial emergency treatment structures, it is equally important to develop dedicated post-acute care concepts and rehabilitation frameworks that seamlessly continue from inpatient treatment. To quantify the need for such structural changes and stabilizations within the broader healthcare system, more comprehensive data is needed beyond just polytrauma cases or preclinical emergency care statistics. Everyday injuries, and even minor injuries, already place a significant daily burden on existing care structures.

Therefore, it is essential to adapt the structures to provide trauma care that is high-quality, efficient, accessible, and modern. This particularly involves the provision of needs-based care structures by appropriately distributing specialized care in trauma centers alongside basic care services in more rural areas, leveraging modern technologies such as telemedicine. The provision of funding to maintain these services is an indispensable prerequisite for nationwide, centralized quality and reliability in trauma surgery care in Germany.

However, not only the structures but also the processes of trauma care are crucial. Emergency care must be managed so that cases are directed to facilities equipped with appropriate services. Only in cases of unclear or significant health risks should emergency treatment be conducted in hospitals, ideally in integrated emergency centers. Furthermore, the public could be supported in distinguishing less urgent and non-life-threatening emergencies through online resources or advisory services, thereby avoiding unnecessary use of trauma care resources.

The separation of different sectors also poses a challenge within trauma care. Scarce personnel can only be flexibly, interdisciplinarily, and interprofessionally deployed if cross-sector treatment and billing are possible. Overcoming sector boundaries is also vital to ensure medical training can occur across sectors and providers. Ultimately, approaches such as cross-sector, regional health budgets could contribute to promoting prevention and health promotion.

While healthcare professionals are increasingly retiring and birth rates remain low, patient complexity and expectations for restoring a livable life well into old age are rising. Simultaneously, potential new recruits approach the field of trauma surgery with hesitation or outright rejection, influenced by legitimate demands for better work-life balance. Mastering the comprehensive patient clientele and the varied emergency and elective aspects of trauma surgery requires extensive experience. Becoming an experienced trauma surgeon demands time and exposure to surgeries and patients – two critical factors that may become insufficient in the future.

Consequently, training and working conditions in trauma surgery should be improved to particularly enhance skills acquisition and enable better compatibility between career and family life. This can be achieved by reducing bureaucracy and documentation burdens, as well as by sensibly relieving trauma surgeons with the help of new professional groups and technologies.

The challenges facing trauma care in Germany are undoubtedly significant. However, this need not be an inevitable fate: we possess the means and methods to meet these challenges. What is now required is the courage and determination to adjust the framework conditions so that we can utilize these resources effectively and thus secure trauma care in the future.

## Limitations

This study has several limitations. First, all quantitative analyses are based on hospital quality reports, ICD-coded inpatient data, and hospital directories up to 2019. As such, our projections to 2030 do not explicitly account for structural disruptions caused by the COVID-19 pandemic, recent inflationary shocks, or the ongoing hospital reform process. The scenarios should therefore be interpreted under the aspect of pre-pandemic baseline projections resulting in as precise as possible forecasts.

Second, the online survey relies on voluntary participation of members of the German Society of Trauma Surgery (DGU). The respondent cohort is characterized by a predominance of senior and chief surgeons and male participants, which may limit generalizability to younger surgeons and under-represented groups. Self-selection and recall bias cannot be excluded.

Third, the expert panel employed a structured workshop format but did not follow a formal Delphi process. The strategic recommendations reflect the consensus of 23 experienced DGU members and are inherently shaped by their professional perspectives and the German healthcare context. While we believe that the triangulation of quantitative data, survey results, and expert judgment enhances the robustness of our conclusions, the transferability to other countries and health systems may be limited and requires adaptation to local conditions.

## Data Availability

All used data is shown in he manuscript and would be availabel on further demand.
